# Everything Is Permitted? People Intuitively Judge Immorality as Representative of Atheists

**DOI:** 10.1371/journal.pone.0092302

**Published:** 2014-04-09

**Authors:** Will M. Gervais

**Affiliations:** University of Kentucky, Department of Psychology, Lexington, Kentucky, United States of America; George Mason University/Krasnow Institute for Advanced Study, United States of America

## Abstract

Scientific research yields inconsistent and contradictory evidence relating religion to moral judgments and outcomes, yet most people on earth nonetheless view belief in God (or gods) as central to morality, and many view atheists with suspicion and scorn. To evaluate intuitions regarding a causal link between religion and morality, this paper tested intuitive moral judgments of atheists and other groups. Across five experiments (*N* = 1,152), American participants intuitively judged a wide variety of immoral acts (e.g., serial murder, consensual incest, necrobestiality, cannibalism) as representative of atheists, but not of eleven other religious, ethnic, and cultural groups. Even atheist participants judged immoral acts as more representative of atheists than of other groups. These findings demonstrate a prevalent intuition that belief in God serves a necessary function in inhibiting immoral conduct, and may help explain persistent negative perceptions of atheists.

## Introduction

Without God and the future life? It means everything is permitted now, one can do anything?

-Dostoevsky, *The Brothers Karamazov*

If you learn about an individual's moral or immoral conduct, what else can you infer about that person's beliefs? For instance, if you learn that an individual kills homeless people for fun, or has consumed human flesh, what else might you guess about him or her? The present experiments evaluate the degree to which people perceive religious belief as a necessary component of morality. To individuals who intuitively assume that morality primarily arises from religion, another person's moral behavior may be seen as diagnostic of his or her religious beliefs—or lack thereof. Across experiments, the present paper tested the degree to which immoral behavior is intuitively seen as a signal of religious disbelief.

### Religion and Morality: Reality and Perception

Is religion the bedrock of morality? On the one hand, religion is linked to a variety of positive outcomes, including prosocial behavior [1]–[4], volunteerism [5], honesty [6]–[7], and an ability to resist temptation [8]–[9]. Religions may have been instrumental in the development of moral communities [10] that foster cooperation. On the other hand, moral judgments rely heavily on intuitions that emerge early in development [11] and may be shared with close primate relatives [11]–[15]. These moral intuitions may suggest the operation of a universal moral grammar [16] that is robust across differences in religion [17]–[18].

Although scientific opinion on the relationship between religion and morality is somewhat ambiguous, popular opinion seemingly is not. Most Americans report that belief in God is an integral component of morality, a sentiment echoed at least as strongly in most countries worldwide [19]. A perceived intimate connection between religion and morality may engender widespread reactions of exclusion [20], distrust [21]–[24], and disgust [25] towards atheists around the world.

An assumed causal relationship between religion and morality has the potential to influence the intuitive assumptions that often underlie stereotyping and prejudice. People readily form intuitive representations of a person's likely group memberships given only minimal information about that person [26]. To the extent that people view morality as deriving from religious belief, then information about a person's moral conduct may be intuitively viewed as diagnostic of that person's religious beliefs. In other words, to an observer who thinks that religion enables people to inhibit immoral behavior, learning that an agent engages in immoral behavior may be sufficient to lead the observer to intuitively infer that the agent is not religious. Thus, reactions to descriptions of immoral behavior can shed light on people's intuitions regarding the role of religious belief in morality.

An intuitive connection between religion and morality may also help explain the prevalence of negative perceptions of atheists. Atheists are routinely excluded in the U.S.A. [20]. In the context of many classic approaches to prejudice and stereotyping, this is a puzzling form of antipathy [21], [23]. Atheists do not constitute a cohesive or powerful group (if, indeed, they can even meaningfully be thought of as a group), and classic intergroup dynamics do not appear to adequately explain negative perceptions of atheists. In addition, perceptions of warmth and competence do not explain why atheists are perceived even more negatively than other groups similar in this regard [23]. Initial research highlights distrust as one key component in anti-atheist prejudice [22]–[23], [27]. The present research, in addition to exploring intuitive perceptions of a religion-morality link, offers to broaden this investigation of distrust of atheists to consider the broader question of whether atheists are distrusted in part because people intuitively assume that atheists in some way lack a perceived necessary component of morality: religious belief.

### General Method

The present experiments evaluated intuitive perceptions of a causal link between religion and morality by utilizing the representativeness heuristic [26], a mental shortcut that biases people's probability judgments. In a classic problem illustrating this heuristic, participants are given a description of Linda, a politically active, single, liberal woman who cares deeply about social justice and asked whether it is more probable that Linda is A) a bank teller, or B) a bank teller who is active in the feminist movement. Although the first option is necessarily the correct answer (feminist bank tellers being only a subset of bank tellers in general), most people commit a conjunction error: that is, they erroneously pick the latter option because they intuitively judge that the description (single, liberal, politically active) is representative of the potential group membership (feminist) implied in the question. However, if participants are instead given a potential group membership that does not fit the description (e.g., if Linda could be a bank teller who is an avid big game hunter), there is less intuitive pull to commit a conjunction error (the Online Supplement includes an empirical demonstration of this difference). Thus, by independently manipulating the contents of the description and the potential group membership implied in the question, researchers can use the rates of conjunction errors for different targets as an index of the degree to which a given description is intuitively viewed as representative of different groups of people [23].

**Figure 1:**
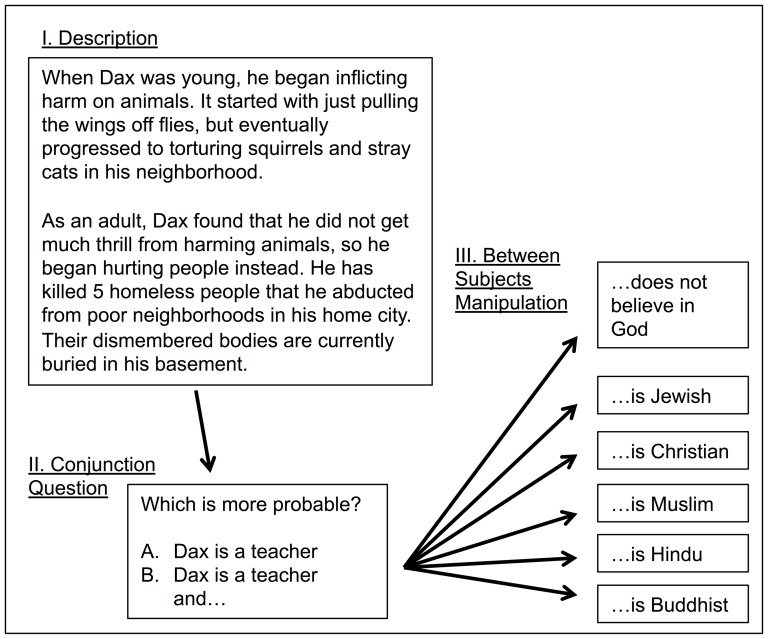
Schematic summary of methods used across experiments, illustrated with the serial killer description used in Experiment 1. Note: For the Buddhist, Hindu, and Muslim conditions, the character was called “a man” rather than “Dax.”.

In five experiments, I presented participants with a description of someone engaging in an action that is often viewed as immoral. Then, between participants, I varied the potential groups to which the person might belong to test the degree to which the immoral act was seen as representative of different groups of people (schematically represented in Figure 1). All five experiments drew samples from adults in the U.S.A. on Amazon Mechanical Turk, an online labor market frequently and productively used in social science research [28]–[29]. Across experiments, I tested moral perceptions of atheists across eight different moral transgressions varying greatly in severity, ranging from relative innocuous (e.g., cheating at cards) to more unconventional (e.g., incest) and even severe (serial murder). In addition, throughout experiments perceptions of atheists were compared to perceptions of a variety of other targets, including Christians, Jewish people, Muslims, Hindus, Buddhists, Whites, Blacks, Asians, Hispanics, Native Americans, and gay men. These experiments therefore present a thorough investigation of intuitive perceptions of a religion-morality link that includes various domains of morality [30], and contrasts atheists to many other religious, ethnic, and cultural outgroups.

## Experiment 1

Experiment 1 presented participants with a description of a person engaging in clearly and unambiguously immoral behavior: the character tortures animals as a child and, as an adult, abducts, kills, and dismembers five homeless people before burying them in his basement. Crucially, Experiment 1 tested the hypothesis that participants would intuitively find a description of an animal torturer and serial murderer to be more representative of atheists than of a variety of different religious groups.

### Participants

Two hundred thirty seven American adults from Mechanical Turk participated in Experiment 1. The participants represented a wide variety of religious backgrounds (full demographics for all experiments are presented in the supporting information: Table S1 and Figure S1 in File S1). I aimed to recruit at least 30 participants per cell, and deliberately oversampled to meet this goal. All sample size decisions were made *a priori*. Three participants failed an Instrumental Manipulation Check [31] and were excluded before any analyses were conducted.

### Procedure

All research was approved by the University of Kentucky Office of Research Integrity. Participants first completed an IRB-approved online consent procedure. Participants read a consent form. After reading the form and having an opportunity to email with any questions, participants checked a box to confirm that they were at least 18 years old, had read and understood the consent form, and agreed to participate. After giving digital consent, participants proceeded to the main survey.

In the main survey, participants were first presented with the following description of a moral transgressor:

*When Dax was young, he began inflicting harm on animals. It started with just pulling the wings off flies, but eventually progressed to torturing squirrels and stray cats in his neighborhood.*

*As an adult, Dax found that he did not get much thrill from harming animals, so he began hurting people instead. He has killed 5 homeless people that he abducted from poor neighborhoods in his home city. Their dismembered bodies are currently buried in his basement.*

Following this description, participants were asked whether it is more probable that the character is A) a teacher, or B) a teacher who XXXXXX, with XXXXXX varied between subjects. XXXXXX was either “does not believe in God” (*N* = 35), “is a Buddhist” (*N* = 39), “is a Christian” (*N* = 38), “is a Hindu” (*N* = 51), “is Jewish” (*N* = 44), or “is a Muslim” (*N* = 30). Experiment 1 referred to the atheist target merely as someone who does not believe in God to mitigate kneejerk negative reactions to the term “atheist.” In the Buddhist, Hindu, and Muslim conditions, the villain was referred to as “a man” rather than “Dax.” (See, however, Experiment 5 and a pilot study in the Online Supplement demonstrating that effects do not seem to be affected by this name difference).

Immediately following the conjunction question, participants had one additional item as an Instrumental Manipulation Check [31] to exclude participants not paying attention to directions. This item included a question about US Presidents, with a drop down menu providing several response choices. However, in the instructions for this item, participants were told to skip the question without leaving a response.

Next, participants proceeded to a different screen that included four syllogistic reasoning problems. These items were included to better conceal the true nature of the conjunction task as merely one in a series of logic puzzles. It should be noted that because the distractor items followed the primary measure of interest in the study, they could not have affected responses. Rather, they were included to reduce the (already slight) risk that performing a task measuring perceptions of a religion-morality link might create social desirability pressures that would compromise participants' own self-reported religious demographics.

Finally, participants completed a series of demographic measures. Participants provided information about age, gender, ethnicity, and religious affiliation, as well as measures of belief in God, political attitudes, and subjective socioeconomic status. Participants rated belief in God, from 0 (God definitely does not exist) to 100 (God definitely exists). Participants rated political attitudes on a dropdown menu, including options Very Liberal, Liberal, Slightly Liberal, Moderate, Slightly Conservative, Conservative, Very Conservative. These responses were coded numerically (1 = Very Liberal, 7 = Very Conservative). For subjective socioeconomic status [32], participants rated their own perceived status on a ladder (from 0–10), relative to the people in the USA who are the worst off (the bottom of the ladder, 0) and the people in the USA who are the best off (the top of the ladder, 10). Finally, participants entered their state of residence and current zip code before being redirected to an online debriefing with instructions on how to redeem their Mechanical Turk payments.

### Results

**Figure 2:**
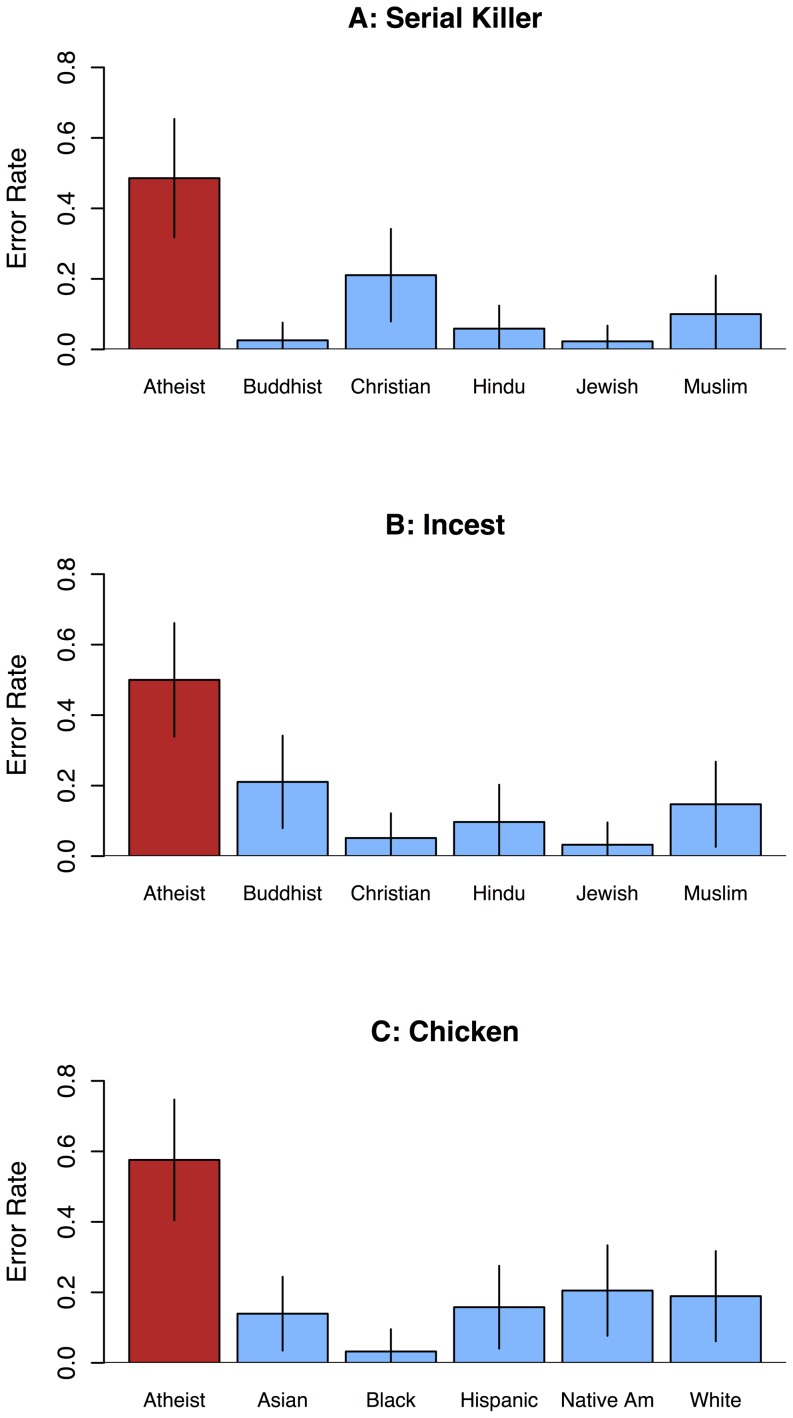
Conjunction error rates (proportion), Experiments 1–3. A) Given a description of serial murder and animal torture, participants were significantly more likely to commit a conjunction error for the atheist target than for any of five religious targets. B) Given a description of consensual incest, participants were significantly more likely to commit a conjunction error for the atheist target than for any of five religious targets. C) Given a description of a man having sex with, then eating, a dead chicken, participants were significantly more likely to commit a conjunction error for the atheist target than for any of five ethnic targets. Error bars represent 95% confidence intervals of the mean.

All analyses were performed in R [33]. Participants were significantly more likely to commit a conjunction error (i.e., picking the “teacher and XXXXXX” option) for targets who do not believe in God (48.6% errors) than for Buddhist (2.6%), Christian (21.1%), Hindu (5.9%), Jewish (2.3%), or Muslim (10.0%) targets, see Figure 2a (Table 1 presents logistic regression results for Experiments 1–3). Participants viewed animal torture and serial murder as representative of atheists, but not of various religious groups.

**Table 1:** Logistic regression summaries, Experiments 1–3.

	OR	Low	High	*p*
**Exp. 1**				
All	10.98	4.79	25.78	2×10^−8^
Buddhist	35.89	6.59	672.03	8×10^−4^
Christian	3.54	1.31	10.28	.015
Hindu	15.11	4.44	70.48	7×10^−5^
Jewish	40.61	7.48	759.40	5×10^−4^
Muslim	8.50	2.43	40.31	.002
**Exp. 2**				
All	8.11	3.69	18.20	2×10^−7^
Buddhist	3.75	1.41	10.72	.01
Christian	18.50	4.72	124.05	2×10^−4^
Hindu	9.33	2.72	43.78	.001
Jewish	30.00	5.52	561.21	.001
Muslim	5.80	1.96	19.95	.002
**Exp. 3**				
All	7.76	3.52	17.56	5×10^−7^
Asian	8.37	2.91	27.16	2×10^−4^
Black	40.71	7.23	768.59	6×10^−4^
Hispanic	7.24	2.50	23.64	4×10^−4^
Nat. Am.	5.26	1.92	15.58	.002
White	5.82	2.06	18.01	.001

For each experiment, results from six logistic regression models are presented, comparing (1) the atheist target to all targets (All), followed by (2–6) comparisons of the atheist target to each other target individually. Odds ratios, as well as upper and lower bounds of a 95% confidence interval of the odds ratio, are presented, along with p-values.

Experiment 1 demonstrated that one particularly vivid example of immorality—serial murder—is seen as representative of atheists. Subsequent studies relied on widely studied examples of people's moral intuitions, drawing upon examples from the work of Haidt and other Moral Foundations Theory researchers [30], [34].

## Experiment 2

Murder presents a strong and clear example of immorality. Yet people often intuitively find other acts immoral even if the acts do not involve harm to others [34]. Experiment 2 followed the procedure of Experiment 1, and tested whether one such seemingly victimless moral violation—consensual incest—was similarly judged as more representative of atheists than of other groups.

### Participants

Two hundred eleven American adults from Mechanical Turk participated in Experiment 2. The participants represented a wide variety of religious backgrounds (full demographics for all experiments are presented in the Online Supplement). As in Experiment 1, I aimed to recruit at least 30 participants per cell, and deliberately oversampled to meet this goal. All sample size decisions were made *a priori*. Three participants failed an Instrumental Manipulation Check [31] and were excluded before any analyses were conducted.

### Procedure

The procedure of Experiment 2 was identical to that used in Experiment 1. Only the contents of the described moral violation differed. Participants read the following description, before completing the rest of the study exactly as in Experiment 1:

*Graeme and his sister were traveling together in France. One night they were staying alone in a cabin near the beach. They decided that it would be interesting and fun if they tried making love. At very least it would be a new experience for each of them. Graeme's sister was already taking birth control pills, but Graeme used a condom too, just to be safe. They both enjoyed it, but they decided not to do it again. They keep that night as a special secret between them, which makes them feel even closer to each other.*

Next, participants completed one of six versions of the conjunction problem, using the same potential religious affiliations used in Experiment 1 (sample sizes: atheist = 38, Buddhist = 38, Christian = 39, Hindu = 31, Jewish = 31, Muslim = 34). The career listed for the villain (i.e., the “bank teller” part of the original Linda Problem) in Experiment 2 was “works in retail.”

### Results

In Experiment 2, participants intuitively judged a description of a man engaging in consensual incest with his sister to be more representative of people who do not believe in God (50.0% errors) than of Buddhist (21.1%), Christian (5.1%), Hindu (9.7%), Jewish (3.2%), or Muslim (14.7%) targets, see Figure 2b and Table 1. As with serial murder and animal torture, participants found a description of someone engaging in consensual incest to be more representative of atheists than of other religious groups.

## Experiment 3

Experiment 3 extended Experiment 2 by testing whether another “victimless” moral violation—necrobestiality—was similarly seen as more representative of atheists than of other groups. Rather than compare atheists to different religious groups, Experiment 3 compared atheists to different ethnic groups.

### Participants

Two hundred twenty one American adults from Mechanical Turk participated in Experiment 3. The participants represented a wide variety of religious backgrounds (full demographics for all experiments are presented in File S1). As in Experiments 1–2, I aimed to recruit at least 30 participants per cell, and deliberately oversampled to meet this goal. All sample size decisions were made *a priori*. Nine participants failed an Instrumental Manipulation Check [31] and were excluded before any analyses were conducted.

### Procedure

The procedure of Experiment 3 was identical to that used in Experiments 1–2. Only the contents of the described moral violation and the potential group memberships differed. Participants read the following description, before completing the rest of the study exactly as in Experiments 1–2:

*On the way home from work, Jack decided to stop at the butcher shop to pick up something for dinner. He decided to roast a whole chicken. He got home, unwrapped the chicken carcass, and decided to make love to it. He used a condom, and fully sterilized the carcass when he was finished. He then roasted the chicken and ate it for dinner alongside a nice glass of Chardonnay.*

Next, participants completed one of six versions of the conjunction problem (sample sizes: atheist = 33, Asian = 43, Black = 31, Hispanic = 38, Native American = 39, White = 37). The career listed for the villain (i.e., the “bank teller” part of the original Linda Problem) in Experiment 3 was “doctor.”

### Results

Participants intuitively judged a description of a man having sexual intercourse with, then cooking and eating, a dead chicken to be more representative of people who do not believe in God (57.6% errors) than of Asian (14.0%), Black (3.2%), Hispanic (15.8%), Native American (20.5%), or White (18.9%) targets, see Figure 2c and Table 1. As with the case of incest presented in Experiment 2, participants intuitively found a description of a man having sex with and eating a dead chicken to be representative of atheists. This effect was not apparent for any of five ethnic group memberships.

## Experiment 4

Experiment 4 extended the findings of Experiments 1–3 in two ways. First, following previous research [22]–[23], Experiment 4 used perceptions of gay men—another cultural outgroup frequently excluded in the U.S.A.—as a strong comparison for perceptions of atheists. Both atheists and gays have concealable identities and are often derogated in explicitly moralistic terms. Yet, negative perceptions of atheists and gays appear to derive from different psychological bases [23], potentially leading moral violations to be viewed as more representative of atheists than of gays. Second, Experiment 4 used a broader range of moral violations, following Moral Foundations Theory [30], [34], which posits five basic themes for moral judgment: harm, fairness, loyalty to the ingroup, obedience to authority, and purity. In Experiment 4, participants were presented with descriptions of people violating each of the five foundations (Harm: ridiculing an obese woman and kicking a dog; Fairness: reneging on reciprocity norms and cheating at cards; Ingroup: renouncing national and family ties; Authority: disrespecting employers and police officers; Purity: eating human flesh; full scenarios are included in the Online Supplement). Experiment 4 thus utilized a 5 (type of moral violation) by 2 (potential atheist target vs. potential gay target) between subjects design.

### Participants

Three hundred twenty seven American adults from Mechanical Turk participated in Experiment 4. The participants represented a wide variety of religious backgrounds (full demographics for all experiments are presented in the Online Supplement). As in Experiments 1–3, I aimed to recruit at least 30 participants per cell, and deliberately oversampled to meet this goal. All sample size decisions were made *a priori*. The final sample sizes across atheist and gay conditions (respectively) were as follows: Harm (34, 34), Fairness (27, 29), Ingroup (39, 34), Authority (35, 33), and Purity (30, 37).

### Procedure

The general procedure of Experiment 4 was identical to that used in Experiments 1–3. Experiment 4 did not include an Instrumental Manipulation Check or distractor syllogisms. Participants read one of five descriptions of a moral transgression (representing violations of each of the five moral foundations). Then, they received a conjunction question with either a potential atheist target (referred to as “an atheist [someone who does not believe in God]”) or a potential gay target. Following this conjunction question, they completed demographics, as in previous experiments. As in Experiment 1, the career listed for the villain (i.e., the “bank teller” part of the original Linda Problem) in Experiment 4 was “teacher.”

### Results

Participants heuristically judged descriptions of all five types of moral violations as more representative of people who do not believe in God than of gay people (see Table 2 for logistic regression summaries). In sum, across different moral foundations, participants found descriptions of a moral transgressor to be more representative of atheists than of gay people. This provides a critical contrast, as it suggests that it is not mere counternormativeness of a group that leads people to intuitively view immorality as representative of that group.

**Table 2:** Logistic regression summaries, Experiment 4.

	Atheist	Gay	OR	Low	High	*p*
**Harm**	47.1	14.7	5.16	1.70	18.04	.006
**Fairness**	33.3	0.0	30.30	3.49	∞	.02
**Ingroup**	38.5	14.7	3.63	1.21	12.48	.03
**Authority**	31.4	6.1	7.10	1.70	48.79	.02
**Purity**	43.3	0.0	57.86	6.99	∞	.006

Results from five logistic regression models are presented, comparing the atheist target to the gay target for each Moral Foundation violation. The % of conjunction errors in atheist and gay conditions, odds ratios, upper and lower bounds of a 95% confidence interval of the odds ratio, and p-values are presented. Note: In the Fairness and Purity conditions, no participants committed conjunction errors with a potential gay target, rendering traditional logistic regression models impossible. Instead, bias-reduced GLM analyses were performed using the brglm package in R.

Although most of these conditions include descriptions of two moral violations, an additional study presented in the Online Supplement demonstrates that identical results are evident if the description only includes one relatively minor moral breach (cheating at cards once). In sum, the results of the present research are not attributable to all perpetrators being described as sadistic (Experiment 1), bizarre (Experiments 2–4), or someone who repeatedly engages in immoral behavior (Experiment 4). Instead, even a description of someone cheating at cards one single time is sufficient to produce the present effects.

Finally, the Online Supplement (Table S2 in File S1) presents additional analyses showing that, largely consistent with previous findings in Moral Foundations research [30], political conservatism significantly predicted conjunction error rates in the authority violation and purity violation conditions for the potential atheist target.

## Experiment 5

Experiments 1–4 revealed that people intuitively assume that the perpetrators of immoral actions do not believe in God. While previous large-scale global polls consistently and unequivocally indicate a perceived link between belief in God and morality [19], this perception may not neatly map onto the actual mechanisms linking religion and morality. Specifically, religions furnish people not only with metaphysical claims about agents present in the world (e.g., gods), they also provide people with communities, replete with specific teachings and norms [10], [18]. Thus, it may be that immoral actions are seen as representative of not only people who do not believe in God (as revealed by previous polls [19] and Experiments 1–4), but also of people who do not belong to a religious moral community.

Experiment 5 sought to extend the present findings to consider the question of whether immorality is viewed as representative of atheists in part because observers infer that an atheist may not belong to a moral community. In other words, Experiment 5 tests the relative contributions of belonging to a religious moral community and belief in God, respectively, to the present effects. Experiment 5 utilized a 2 (belief in God: yes vs. no) by 2 (moral community: member of a church vs. not a member of a church) between subjects design to directly address the possible contributions of perceived belongingness to a community for the present effects.

### Participants

One hundred fifty one American adults from Mechanical Turk participated in Experiment 5 (full demographics for all experiments are presented in File S1). Again, I aimed to recruit at least 30 participants per cell, and deliberately oversampled to meet this goal. All sample size decisions were made *a priori*. Four participants failed an Instrumental Manipulation Check [31] and were excluded before any analyses were conducted.

### Procedure

The general procedure of Experiment 4 was identical to that used in previous experiments. Participants read the serial killer description used in Experiment 1 (the villain was called “a man” rather than given a name because throughout Experiments 1–4, the villain's name had no discernible effect on the outcomes). Following this description, participants were asked whether it is more probable that the character is A) a teacher, or B) a teacher who XXXXXX, with XXXXXX varied between subjects to reflect a 2 (belief in God) by 2 (religious moral community) manipulation. XXXXXX was either “does not belong to any church and does not believe in God” (*N* = 43), “belongs to a church but does not believe in God” (*N* = 39), “does not belong to any church but believes in God” (*N* = 33), or “belongs to a church and believes in God” (*N* = 32). This design thus enabled a direct test of the contributions of belief in God and belonging to a religious moral community. Following the conjunction task, participants completed an Instrumental Manipulation Check [31], as in other studies. Then they recorded only age and gender as demographics.

### Results

To test the relative contributions of belief in God and belonging in a religious moral community to the present effects, I first conducted a 2 (belief in God: yes vs. no) by 2 (moral community: member of a church vs. not a member of a church) binary logistic regression. This analysis revealed a significant main effect of belief in God, *b* = 1.10, *se* = .56, *p* = .048, but no main effect of moral community (*b* = −.04, *se* = .64, *p* = .95) and no interaction (*b* = −.12, *se* = .78, *p* = .88) (Full pattern of results: Atheist, Yes church = 41% errors; Atheist, No church = 37%; Believer, Church = 19%; Believer, No church = 18%). Given the lack of a significant interaction, I performed two separate analyses exploring the effects of belief in God (collapsing across moral community conditions) and moral community (collapsing across belief in God conditions), respectively. The first analysis revealed that—regardless of described belongingness to a religious moral community—participants judged serial murder as representative of people who do not believe in God, *OR* = 2.83, 95% CI: 1.34 to 6.28, *p* = .008. In contrast, collapsing across belief in God, not belonging to a religious moral did not significantly increase conjunction error rates, *OR* = .91, 95% CI: .45 to 1.84, *p* = .78. In sum, when given a description of someone engaging in immoral behavior, participants readily and intuitively assume that the villain does not believe in God, but apparently make no inferences regarding whether or not the villain is a member of a religious moral community. While moral communities may be instrumental in any actual link between religion and moral outcomes [10], moral communities seem to have little bearing on lay perceptions of such a link, an interesting divergence discussed in more detail in the General Discussion.

## Aggregate Atheist Analyses

Finally, I investigated atheists' own intuitive moral perceptions of atheists. Pooling across experiments for which religiosity data were collected (Experiments 1–4), I conservatively isolated only those participants (*N* = 163) who both self-identified as atheists and who rated their belief in God at 0. I conducted a logistic regression model predicting conjunction error rates from potential group membership (atheist target vs. all other targets). This analysis revealed that even atheist participants viewed immorality as significantly more representative of atheists than of other people, *OR* = 4.47, 95% CI: 1.62 to 12.66, *p* = .004. Even atheists seem to share the intuition that immoral acts are perpetrated by individuals who don't believe in God. This suggests an intuitive association between morality and belief in God is not an exclusively religious intuition (see Table S3 in File S1 for analyses testing the moderating effects of participant belief in God across experiments).

This aggregate analysis is conceptually analogous to performing a meta-analysis on the atheist participant effects from each study. However, the aggregate is more efficient and does not require four separate analyses (one for each study) that are likely each grossly underpowered. In the reported analysis, I included three dummy codes for study number, to simultaneously account for between-study differences. Inferences are identical if these dummies are not included.

## General Discussion

In sum, when reading a description of someone committing an immoral act, participants readily and intuitively assumed that the person was an atheist. Combined, these results demonstrate that Americans (even atheist Americans) intuitively assume that belief in God somehow inhibits people from engaging in immoral behavior. Interestingly, Experiment 5 suggests that people are skeptical of atheist morality specifically because atheists do not believe in God, not merely because atheists are not members of religious moral communities. Strikingly, these results were apparent even among MTurk participants, who tend to be less religious, on average, than Americans in general (see demographics presented in File S1). Although the present experiments only utilized American samples, previous polls [19] indicate that an association between religious belief and morality is by no means an exclusively American trend. Nonetheless, future research should seek to replicate the present studies in diverse populations worldwide. To this end, an initial pilot cross-cultural investigation reported in the Online Supplement replicated the effects of Experiment 1 among participants in India.

Importantly, previous research [23] using the same experimental procedure demonstrates that these effects do not represent a general effect whereby any negatively valenced description is viewed as representative of atheists. The present findings, combined with previous research using this exact experimental paradigm [23], instead suggest that it is specifically immoral negative actions that are seen as representative of atheists, consistent with other evidence suggesting that many view belief in God as a prerequisite for morality [19].

### Moving Forward

People's intuitive perceptions of a necessary link between religion and morality can potentially serve as an interesting contrast to some recent research demonstrating that moral judgments draw heavily upon innate intuitive responses. Although the issue of the degree which morality is innate is far from closed, it is interesting to speculate about how learning about research suggesting an innate component of morality might affect perceptions of atheists. Specifically, it is possible that—regardless of the degree to which morality actually derives from core intuitions subsequently elaborated through cultural learning [34]—participants who read about an innate core foundation of morality might form different moral perceptions of others. If true, then exposure to scientific arguments regarding the developmental [11], phylogenetic [12], or neural [35]–[36] underpinnings of common moral intuitions might alleviate morality-driven antipathy towards atheists. Indeed, some popular treatments of this research [13] explicitly make the point that the research in question has implications for the role of religion in morality.

These experiments also leave open the question of which aspects of religious belief people think inhibits immorality. On the one hand, religious traditions often include explicit teachings regarding what is permitted and forbidden (e.g., the Ten Commandments, the Buddhist Noble Eightfold Path). People may intuitively associate immorality with atheists because of uncertainty regarding whether or not atheists know which acts are immoral. On the other hand, it could be that people view atheists as capable of telling right from wrong, but lacking an external motivational structure incentivizing morality (e.g., heaven) and disincentivizing immorality (e.g., hell).

Finally, the present findings may shed light on the psychological factors that contribute to prevalent prejudice against atheists. Atheism is a concealable stigma, and atheists do not constitute a cohesive, coordinated, or conspicuous group; nonetheless they are among the least accepted people in America [20]. This pattern is initially puzzling, and makes little sense in the context of many approaches to prejudice and stereotyping that stress the role of intergroup dynamics or perceptions of warmth and competence [21], [23]. However, the present studies suggest that inferences made about individuals, rather than perceptions of group characteristics, likely underpin anti-atheist prejudice. Atheists' individual and collective inconspicuousness may leave people uncertain about what exactly atheists are like. However, if people readily and intuitively assume that the perpetrators of immoral acts are atheists, then this may generate group-level stereotypes of widespread atheist immorality, leading to distrust [23] and disgust [25] of atheists.

### Religion and Morality: Reality and Perception Revisited

Much ink has been spilled debating the role of religion in morality. Although current research seems to suggest that at least some core building blocks of morality do not depend overly much on religious enculturation [11], [12]–[17], it is likely that a species as thoroughly cultural as *Homo sapiens* nonetheless experiences elaboration of any innate moral sentiments [11] through cultural learning. Inasmuch as this is true, religion likely does exert some influence on morality in at least two ways. First, one mechanism through which religion may actually influence morality is by the creation of moral communities [10]. In this sense, individuals will adopt moral norms from those in their surrounding communities, via well-elaborated cultural learning processes [37]–[40]. A second mechanism linking religion to morality stems from a wealth of recent research finding that belief in, and reminders of, God can influence moral outcomes [2]–[4], [8]–[9]. These two mechanisms are not mutually exclusive, and likely both operate in concert. Indeed, the cultural success of many religions likely stems from them doing many things well, rather than from a single mechanism. Likely, this is also the case in the moral domain.

While any scientific consensus on the actual relationship between religion and morality seems likely to be inevitably complex, nuanced, and multifaceted, the present studies (as well as previous worldwide polling: [19]) provide initial evidence that lay perceptions of a religion-morality link focus specifically on belief in God, rather than community. Across studies, immoral deeds were heuristically judged as representative of individuals who don't believe in God, even though the stimuli gave no specific information about moral communities. Further, Study 5 directly tested the contributions of belief in God and belonging to a religious moral community, and found no support for the possibility that lay perceptions of a religion-morality link are consonant with research suggesting a prominent role for community [10]. Interestingly, while available evidence suggests prominent roles for both community and faith in shaping moral outcomes, lay perceptions may overestimate the role of faith while underestimating the role of community in shaping morality.

### Coda

Recently, successful lines of research have emerged, independently investigating the evolutionary and cognitive origins and consequences of both religion [1]–[2], [41]–[43] and morality [11]–[13], [29], [34]–[35]. The present paper seeks to integrate these perspectives by focusing on perceptions of religion's role in enabling and facilitating morality. Religions may have been instrumental in the cultural evolution of large-scale human cooperation [2] by binding people into moral communities [10], [44]. However, a moral community is defined as much by those included within it as by those excluded from it. These experiments reveal one potentially pernicious outcome of this exclusion: intuitive associations of immorality with disbelief in God.

## Supporting Information

File S1 Contains full stimuli sets, participant demographics, analyses on political affiliations and religiosity, as well as three additional pilot studies. Figure S1, Density plot of belief in God across Experiments 1–4. Table S1, Participant demographics across all experiments. Table S2, Logistic regression summary for political attitudes predicting conjunction error rates for atheist targets in Experiment 4. Table S3, Logistic regression summary for belief in God predicting conjunction error rates for atheist targets across Experiments 1–4.
(DOCX)
