# Supplementary material for: Everything Is Permitted? People Intuitively Judge Immorality as Representative of Atheists
Source: PLoS One. 2014 Apr 9;9(4):e92302. doi: 10.1371/journal.pone.0092302 (PMC3981659; doi:10.1371/journal.pone.0092302)
Supplement: File S1 — Contains full stimuli sets, participant demographics, analyses on political affiliations and religiosity, as well as three additional pilot studies. Figure S1, Density plot of belief in God across Experiments 1–4. Table S1, Participant demographics across all experiments. Table S2, Logistic regression summary for political attitudes predicting conjunction error rates for atheist targets in Experiment 4. Table S3, Logistic regression summary for belief in God predicting conjunction error rates for atheist targets across Experiments 1–4. (DOCX) [file pone.0092302.s001.docx]

**Everything is Permitted?**

**People Intuitively Judge Immorality as Representative of Atheists**

Supporting Information

Table of Contents

Supporting Methods2

Complete Stimuli Sets2

Full Participant Demographics4

Supporting Analyses6

Political Beliefs: Experiment 46

Religiosity: Experiments 1-48

Linda Problem Pilot Study9

India Pilot Study10

Single Violation Pilot Study12

**Supporting Methods: Additional Methodological Details**

**Full Stimuli Sets**

With the exception of the serial killer example used in Experiment 1, all stimuli were adapted from materials previously used by Haidt, Graham, and other members of the Moral Foundations Theory team. Stimuli for Experiments 1-3 are presented in the main manuscript.

**Experiment 4**

**Harm.** Russell was on the bus on his way home from a long day at work. An overweight woman got on the bus. Rather than give up his seat for the woman, Russell made a number of cruel remarks to her about her appearance. After getting off the bus, Russell was walking to his apartment. A stray dog walked up to him to beg for food and Russell kicked it in the head, hard.

**Fairness.** Brad just moved into a new apartment. A coworker, Jan, helped Brad move his belongings. One day, Jan emailed Brad to see if Brad would help Jan move into a new apartment. Brad ignored the email and did not help Jan. Brad also enjoyed playing poker at a local bar. When he was playing with people he did not know well, Brad would usually cheat in order to win money.

**Ingroup.** Lesley grew up in the USA. After university she spent a summer backpacking around Europe. In Europe, she would often pretend that she was Canadian and say derogatory things about the USA. She also rooted against the US team during the Olympics that year. This caused some conflicts with her family, so she cut off all ties with them for one year.

**Authority.** Drew did not like her boss. One day, her boss was scolding Drew for missing a deadline. Drew got frustrated and flipped her boss off as soon as the boss turned around. Later that day, Drew was walking home. The police had a barricade set up on one block for a parade the next day. When a police officer asked Drew to find another route home, Drew said, “Why won’t you f#$%@! pigs just leave me alone?” and walked through the barricade anyways.

**Purity.** Catherine works in a medical school pathology lab as a research assistant. The lab prepares human cadavers that are used to teach medical students about anatomy. The cadavers come from people who had donated their body to science for research. One night Catherine is leaving the lab when she sees a body that is going to be discarded the next day. Catherine was a vegetarian, for moral reasons. She thought it was wrong to kill animals for food. But then, when she saw a body about to be cremated, she thought it was irrational to waste perfectly edible meat. So she cut off a piece of flesh, and took it home and cooked it. The person had died recently of a heart attack, and she cooked the meat thoroughly, so there was no risk of disease.

**Participant Demographics**

**Table S1.** Participant demographics across all experiments.

|  | **Exp. 1** | **Exp. 2** | **Exp. 3** | **Exp. 4** | **Total** |
| --- | --- | --- | --- | --- | --- |
| **Gender (% Female)** | 33.8 | 41.7 | 38.5 | 38.5 | 37.9 |
| **Age** |  |  |  |  |  |
| Mean | 28.4 | 31.9 | 30.0 | 30.2 | 30.5 |
| SD | 8.8 | 11.3 | 11.3 | 10.0 | 10.37 |
| Range | 18-65 | 18-71 | 18-81 | 18-65 | 18-81 |
| **Religious Affiliation (%)** |  |  |  |  |  |
| Catholic | 13 | 8 | 16 | 13 | 13 |
| Baptist | 6 | 9 | 4 | 10 | 8 |
| Other Christian | 14 | 23 | 16 | 16 | 17 |
| Buddhist | 3 | 4 | 1 | 2 | 2 |
| Muslim | 2 | 0 | .5 | 1 | 1 |
| Jewish | 3 | 1 | 1 | 2 | 2 |
| None | 11 | 14 | 13 | 13 | 13 |
| Atheist | 30 | 18 | 23 | 18 | 22 |
| Agnostic | 18 | 23 | 22 | 22 | 21 |
| Other | 2 | 2 | 2 | 3 | 2 |
| **Belief in God (0-100)** |  |  |  |  |  |
| Mean | 35.6 | 45.5 | 38.8 | 43.2 | 40.8 |
| SD | 41.2 | 42.4 | 42.7 | 42.6 | 42.35 |
| Range | 0-100 | 0-100 | 0-100 | 0-100 | 0-100 |
| Median | 10 | 40 | 10 | 25 | 20 |
| **Ethnicity (%)** |  |  |  |  |  |
| White/Caucasian | 78 | 82 | 85 | 78 | 80 |
| African American | 5 | 4 | 5 | 4 | 5 |
| Hispanic | 3 | 2 | 3 | 6 | 4 |
| Native American | 1 | 1 | 1 | 1 | 1 |
| Asian | 12 | 10 | 6 | 9 | 9 |
| Mixed | 0 | 1 | 0 | 1 | .3 |
| Other | 1 | 1 | 0 | 2 | 1 |
| **Political Attitudes (1-7)** |  |  |  |  |  |
| Mean | 3.1 | 3.5 | 3.0 | 3.2 | 3.2 |
| SD | 1.5 | 1.6 | 1.5 | 1.6 | 1.5 |
| Range | 1-7 | 1-7 | 1-7 | 1-7 | 1-7 |
| Median | 3 | 3 | 3 | 3 | 3 |
| **Subjective SES (0-10)** |  |  |  |  |  |
| Mean | 3.9 | 3.8 | 3.7 | 4.0 | 3.9 |
| SD | 1.6 | 1.6 | 1.7 | 1.6 | 1.6 |
| Range | 0-8 | 0-8 | 0-8 | 0-9 | 0-9 |
| Median | 4 | 4 | 4 | 4 | 4 |

**Figure S1.** Density Plot of Belief in God. Rated from 0 (God definitely does not exist) to 100 (God definitely exists). Data pooled from all participants who provided belief in God information across Experiments 1-4.

**Additional Analyses: Politics in Experiment 4**

One of the most notable findings in Moral Foundations Theory is that liberals and conservatives tend to place different amounts of emphasis on the five moral foundations, with liberals and conservatives alike viewing “individualizing” foundations (Harm and Fairness) as morally relevant, but conservatives also valuing “binding” foundations (Ingroup, Authority, and Purity). Thus I conducted a series of analyses to test the degree to which political attitudes predict conjunction error rates for the atheist target across each condition in Experiment 4.

To do so, I isolated only those participants in conditions with a potential atheist target. Next, I performed five logistic regression analyses predicting conjunction error rates for each moral foundation violation from political beliefs. Because the political attitude item was scored ordinally (with seven choices ranging from “Very Liberal” to “Very Conservative”), Odds Ratios thus refer to the error rate change associated with a single unit shift to the right on the political spectrum (e.g., equivalent to, for example, the shift from “Liberal” to “Slightly Liberal,” the shift from “Moderate” to “Slightly Conservative,” or the shift from “Conservative” to “Very Conservative”). Finally, I re-performed the same series of analyses including belief in God as a covariate (both sets of analyses are in Table S2).

Largely consistent with previous findings in Moral Foundations Theory, political attitudes significantly predicted the degree to which participants found violations of Authority and Purity foundations as representative of atheists. Interestingly, however, political attitudes did not predict conjunction error rates for Ingroup violations, independently or when controlling for belief in God.

**Table S2.** Logistic regression summary for political attitudes (higher values = more conservative) predicting conjunction error rates for atheist targets in Experiment 4.

|  | **Odds Ratio** | **Low CI (2.5%)** | **High CI (97.5%)** | ***p*** |
| --- | --- | --- | --- | --- |
| **No covariates** |  |  |  |  |
| Harm | 1.74 | .99 | 3.63 | .09 |
| Fairness | 1.77 | .88 | 4.09 | .13 |
| Ingroup | 1.25 | .83 | 1.93 | .29 |
| Authority | 2.02 | 1.22 | 3.83 | .01 |
| Purity | 2.36 | 1.33 | 5.18 | .01 |
| **Controlling for Belief in God** |  |  |  |  |
| Harm | 1.64 | .76 | 4.37 | .24 |
| Fairness | 1.71 | .81 | 4.05 | .18 |
| Ingroup | 1.21 | .78 | 1.94 | .39 |
| Authority | 2.01 | 1.17 | 3.90 | .02 |
| Purity | 2.41 | 1.27 | 5.86 | .02 |

**Additional Analyses: Religiosity Across Experiments**

Across experiments (with the exception of Experiment 5), I collected data on participant religiosity. To explore the degree to which participant religiosity moderates the effects presented in the main manuscript, I conducted a series of logistic regressions predicting conjunction error rates for atheist targets by belief in God (standardized) within each experiment. The Odds Ratio thus refers to the error rate change associated with a single standard deviation increase in belief in God (see Table S3).

**Table S3.** Logistic regression summary for belief in God predicting conjunction error rates for atheist targets across Experiments 1-4.

|  | **Odds Ratio** | **Low CI (2.5%)** | **High CI (97.5%)** | ***p*** |
| --- | --- | --- | --- | --- |
| **Experiment 1** | 1.93 | .96 | 4.19 | .07 |
| **Experiment 2** | 1.34 | .66 | 2.81 | .41 |
| **Experiment 3** | 1.68 | .81 | 3.71 | .18 |
| **Experiment 4** |  |  |  |  |
| Harm | 4.92 | 1.99 | 16.20 | .002 |
| Fairness | 1.43 | .60 | 3.60 | .42 |
| Ingroup | 1.19 | .62 | 2.30 | .60 |
| Authority | 1.49 | .73 | 3.21 | .28 |
| Purity | 1.71 | .76 | 4.12 | .20 |

**Linda Problem Pilot Study**

I conducted a pilot study to test whether conjunction error rates are sensitive to the different target groups applied to a given description. To do so, I used the classic “Linda Problem” originally presented by Tversky and Kahneman. Participants (*N* = 62, 44% female, mean age = 28.1) read the following description: “Linda is 31 years old, single, outspoken and very bright. She majored in philosophy. As a student, she was deeply concerned with issues of discrimination and social justice, and also participated in antinuclear demonstrations.” Following this, participants were asked to judge whether it is more probable that Linda is either A) a bank teller, or B) a bank teller who is XXXXXX. Between subjects, I manipulated XXXXXX. Half of the participants received the traditional Linda Problem option of “a bank teller who is active in the feminist movement” and the other half received an alternate option of “a bank teller who is an avid big-game hunter.”

A logistic regression model revealed that, as expected, participants were more likely to commit a conjunction error in the “feminist” condition (82% errors) than in the “big game hunter” condition (0% errors), *Odds Ratio^[[1]](#footnote-1)^* = 249.62, 95% CI: 28.43, ∞, *p* = .0003. Thus, conjunction errors reflect an intuitive linkage between the description provided and specific target groups.

**India Pilot Study**

As an initial attempt to evaluate the findings reported in the main manuscript cross-culturally, I used Mechanical Turk to recruit a sample of participants from India. As in the main experiments, I targeted at least 30 participants per cell. In previous experiments conducted with Indian Mechanical Turk workers, I find considerably higher rates of inattentiveness to instructions using an Instrumental Manipulation Check among Indian MTurkers. As a result, I again deliberately oversampled participants, targeting 100 participants. Of these, 38 failed the check and were omitted from analyses. Thus, I analyzed data from 62 participants (27% female; mean age 30.9; Ethnicity: 88.3% South Asian, 11.7% East Asian; Religion: 66.1% Hindu, 25.7% Christian, 6.5% Muslim, 1.6% Agnostic). Notably, these participants were much more religious than those recruited from American Mechanical Turk samples (mean belief in God or gods = 91.16).

For this pilot study, I presented participants with a modified version of the description used in Experiment 1. Specifically, I removed a reference to torturing squirrels, since squirrels are viewed as sacred within some Hindu sects. Thus, the description used was as follows:

*When a man was young, he began inflicting harm on animals. It started with just pulling the wings off flies, but eventually progressed to torturing stray cats and other animals in his neighborhood.*

*As an adult, the man found that he did not get much thrill from harming animals, so he began hurting people instead. He has killed 5 homeless people that he abducted from poor neighborhoods in his home city. Their dismembered bodies are currently buried in his basement.*

Following this description, I asked participants whether it is more probable that the man was either A) a teacher, or B) a teacher and XXXXXX, with XXXXXX manipulated between participants. For 32 participants, XXXXXX was “does not believe in any gods.” For 30 participants, XXXXXX was “is a religious believer.”

Replicating the effects of Experiment 1, participants were significantly more likely to commit a conjunction error for a potential atheist target (72% errors) than for a potential religious target (30% errors), *OR* = 5.96, 95% CI: 2.06, 18.77, *p* = .001. Indian participants, like their American counterparts, intuitively viewed animal torture and serial murder as more representative of atheists than of religious believers. This pilot study yields initial evidence that a propensity to view belief in God(s) as necessary for morality is not an exclusively American phenomenon. That said, it would be well worth attempting replications in more locations worldwide, including in countries with markedly lower rates of overall religiosity (e.g., the Netherlands, China, New Zealand, Finland, etc.).

**Single Violation Pilot Study**

I conducted a pilot study to test whether the conjunction error rates presented in Experiment 4 were primarily caused by presenting double (rather than single) moral violations. These double violations may have made the villain seem like a habitually immoral psychopath. Thus, this pilot study addresses whether instances of immoral conducted, rather than perceived psychopathy, led participants to intuitively view the character as an atheist.

For this pilot study, I presented participants with a modified version of the Fairness description used in Experiment 4. Specifically, I modified this description so that it only includes a single moral violation. Thus, the description used was as follows:

*A man enjoys playing poker at a local bar. One night, he was playing with people he did not know well, and the man cheated in order to win money.*

Following this description, I asked participants whether it is more probable that the man was either A) a teacher, or B) a teacher and XXXXXX, with XXXXXX manipulated between participants. For 33 participants, XXXXXX was “does not believe in God.” For 35 participants, XXXXXX was “is a religious believer.”

A logistic regression model revealed that, as expected, participants were more likely to commit a conjunction error in the “atheist” condition (45% errors) than in the “believer” condition (17% errors), *Odds Ratio* = 4.03, 95% CI: 1.37, 13.10, *p* = .01. Thus, this pilot study revealed no evidence that conjunction errors in Experiment 4 resulted from the presentation of multiple moral violations.

1. Analyses relied on a bias corrected GLM (the brglm package in R) that can accommodate the 0% error rate in the big game hunter condition. [↑](#footnote-ref-1)
